# Dynamics of parasympathetic activity in violent incarcerated offenders before, during, and in recovery from an emotional inhibition task

**DOI:** 10.1038/s41598-022-10872-y

**Published:** 2022-05-03

**Authors:** Julie Palix, Steven M. Gillespie, Milena Abbiati, Ahmad Abu-Akel

**Affiliations:** 1 Research Unit of Legal Psychiatry and Psychology, Lausanne University Hospital, Route de Cery 1, Lausanne, 1008 Prilly, Switzerland; 2 Department of Primary Care and Mental Health, University of Liverpool, Liverpool, UK; 3 Institute of Psychology, University of Lausanne, Lausanne, Switzerland; 4 School of Psychological Sciences, University of Haifa, Haifa, Israel

**Keywords:** Neuroscience, Psychology, Health care, Medical research

## Abstract

Dynamics of the autonomic nervous system (ANS) are hypothesized to play a role in the emergence of interpersonal violence. In the present study, we examined continuous activities of the inhibitory parasympathetic pathway of the ANS through the root mean square of successive differences between normal heartbeats (RMSSD) in 22 male offenders who committed interpersonal violence and 24 matched controls from the general population across three successive phases: resting baseline, while performing an emotional Go/No-Go task, and post-task recovery. Results showed that across the three phases, the offender group presented lower RMSSD at baseline (*p*_*FDR*_ = .003; Cohen’s d =  − 1.11), but similar levels during the task, attributed to a significant increase in their RMSSD level (*p*_*FDR*_ = .027, Cohen’s *d* =  − 1.26). During recovery, while no distinction between the two groups was found, both groups showed signs of recovering toward baseline values. These findings suggest that violent incarcerated offenders can flexibly engage parasympathetic resources to meet environmental challenges. This underscores the necessity of considering parasympathetic dynamics and its respective mobilization/flexibility to better understand ANS profiles underlying interpersonal violence as well as its potential utility in designing more tailored interventions.

## Introduction

Several studies of interpersonal violence have linked Autonomic Nervous System (ANS) activity at rest and in response to threat or provocation with relationally aggressive behaviors (for reviews see^1,2^. The ANS consists of two antagonistic pathways: the parasympathetic and sympathetic systems (PNS and SNS, respectively). The PNS acts as a brake on the intrinsic rate of the heart to respond rapidly to changing metabolic demands, whether from emotional arousal or from a simple posture change^3–6^. Its involvement is fast to allow a rapid inhibition of the predominant alarm-type, flight-or-fight, SNS activity^7,8^. Thus, heightened PNS activation promotes restorative functions and allows social adaptability and psychological flexibility, while weakened PNS activity is indicative of psychiatric conditions or poor self-regulatory control^3,9,10^. Accordingly, fluctuations in PNS activity—typically measured via continuous recordings of the successive inter-beat intervals of the heart, also called heart rate variability (HRV)^9,11,12^—is of particular interest to understand the mechanisms underpinning behavioral and emotional regulation^3,4^, and putatively, in understanding the loss of control or panic that could occur in episodes of violence^13–16^.

HRV research concerned with the psychophysiological mechanisms underlying violent behaviour has mainly focused on the resting state, measured over a 5-min period of calm^17–19^. While this body of research has consistently shown an association between attenuated levels of PNS at rest (low HRV) and high levels of traits hostility, anger, aggression, and weaker fear extinction^16,20–22^, studying HRV beyond the at-rest phase might provide important new insights about violent behavior. For example, a recent study highlighted that the inclusion of HRV during a negative mood induction task improved the prediction of violent recidivism in young adults^23^.

The importance of characterizing the dynamics of HRV (i.e., during Rest, Reactivity, and Recovery) is further highlighted by a recent theoretical account, which advocates for the need to examine HRV through these different phases, in order to more fully understand how efficiently self-regulatory resources are mobilized and used^24^. In their introduction of The Vagal Tank Theory, Laborde et al. (2018) use the metaphor of the *tank* to represent the self-regulatory resources at one’s disposal during *Rest*, which are depleted in *Reactivity* to an event (such as in completing a task) and then replenished to baseline levels post-event during *Recovery*, in order to face any other upcoming demanding event. According to this theory, the magnitude of the decrease between the baseline and the active phase would correspond to the metabolic demand required by the task, and is expected to be small in, for example, the case of sustained attention^25^ or simple reaction tasks^5^, and near full during a fight or flight response^26,27^. However, some increase in vagal control (increased HRV) from rest to task-related mobilization cannot be ruled out, especially in tasks requiring emotional resources^28^ or empathic skills^29,30^.

Little is known about HRV reactivity (from baseline to task), and recovery post-event in violent offenders. In this study, we examine the PNS dynamics in individuals who have already committed a serious act of interpersonal violence, and how this might inform strategies for the development of self-regulation abilities in this population. Specifically, the current study examines changes in HRV when presented with a cognitively demanding task, how these changes are associated with differences in behavioral performance, and how these resources recover post-event. For the task phase, we chose the emotional Go/No-Go task, which requires individuals to discriminate stimuli of different emotional valences as well as to inhibit a prepotent response^31^. Since it involves processes that have been linked to changes in different frequency bands of HRV^4,28^, this task can highlight differences in autonomic flexibility when challenged. Accordingly, the current study examines the functional dynamics of HRV (PNS activity) during alternating phases of rest, reactivity to an emotional Go/No-Go task, and recovery in violent offenders compared to matched controls. Based on results from previous studies^20,32–34^, and drawing on the predictions of the Vagal Tank Theory^24^, we predicted that the offender group would show low levels of resting HRV, indicative of poorer self-regulatory capacities. Specifically, we predict that the offender group will show (a) reduced HRV at rest compared to controls, and (b) limited reactivity from the baseline to task, and (c) less efficient recovery during the post-task phase. Moreover, because of lower expected HRV, and associated problems in response inhibition and self-regulation, we expect the offender group to underperform the control group in the Go/No-Go task. For the control group, HRV at rest is expected to attenuate during activity while performing the task to accommodate the mental effort needed^5,35^, and a restoration of baseline levels during the recovery phase.

## Material and methods

### Participants

The study sample consisted of 22 incarcerated male offenders (mean age = 39.27 (SD = 12.12) years, range 24–72) and 24 male non-offender controls from the general population (mean age = 32.46 (SD = 12.47) years, range 19 to 58), matched in terms of age, body mass index (BMI) and education (all *ps* > 0.05). The average time spent in custody by offenders at the time of the experiment was 5.95 (SD = 4.68) years. The mean length of their prison sentence was 11.88 (SD = 7.03) years. Over half of the participants in the offender group had been convicted of homicide (54%, 12/22), followed by homicide attempt (32%, 7/22) and other forms of violence (14%, 3/22). Time between crime and testing was about 7.6 years (SD = 7.3). Most of them knew their victim (68%, 15/22), and acted without premeditation (68%, 15/22). According to psychiatric or medical referent records, 45% (10/22) of the sample had previously been diagnosed with personality disorder, 23% (5/22) with psychosis, and 32% (7/22) without any major psychiatric problems. Over half of the sample (55%, 12/22) were Swiss nationals, 36% (8/22) from the EU, and 9% (2/22) outside the EU.

#### Inclusion criteria

Offenders were all male, French speakers, at least 18 years old, and had committed one or more acts of interpersonal violence. We excluded participants who had committed other types of violence (e.g., sexual or against property), were awaiting trial, or could not be tested due to health problems. The testing took place in a dimly lit, quiet room, alone with the experimenter, with a prison officer outside the door following prison authority’s protocol.

We recruited the controls by approaching participants during the mandatory recruitment days of the Swiss army and through public announcements: 75% (18/24) were Swiss nationals, and 25% (6/24) from the EU. We tested participants with the same protocol as the offender group, in a dimly lit, quiet room, alone with the experimenter, either at the army trainee garrisons (6/24 participants), at the university hospital (13/24 participants), or at their domicile (5/24 participants). Participation was voluntary. Participants received 40 CHF compensation, except for the young army recruits, at the request of the Swiss army administration. As confirmed in the personal interview, none of the controls reported a criminal record for a violent offence against another person.

### Procedure

We installed and comfortably positioned an electrocardiogram (ECG) belt in a closed, dimly lit room. Following a short stabilization period, a 5 min baseline ECG—following the guidelines of the Task Force^9^—was obtained first with eyes-opened. The ECG recording continued while participants performed the task (4 min on average), and then for an additional 5 min, at rest, with eyes-opened. The whole session lasted about an hour including the instructions period as well as the installation and removal of the belt. All participants, offenders and controls, provided their signed informed consent before participation. The study was carried out in accordance with the World Medical Association declarations of Helsinki, and approved by the Ethics Committee of the University of Lausanne (CER-VD 58/14).

#### HRV acquisition and analysis

We acquired HRV using a wireless ECG (Equivital system, Cambridge, UK), and processed with the Kubios HRV Premium Software, Version 3.0.2 (Kubios Oy 2016–2019, https://www.kubios.com, Kuopio, Finland) to transform the inter-beat variability into an estimate of PNS activity. HRV values are measured and reported using the time domain RMSSD (Root Mean Square of Successive Differences, in milliseconds)^9,36^, with higher values representing stronger parasympathetic activity. Norms for RMSSD in the general population are reported to average 42 ms ± 15^12^. The RMSSD values were computed over three phases: (1) baseline, (2) task, and (3) recovery for both samples, independently.

#### Task

We employed a shortened version of the emotional Go/No-Go task^37^, to accommodate time constraints for testing in the prison environment. The task was administered using E-Prime version 2.0 (Psychology Software Tools, Inc., Pittsburgh, PA), and consisted of two blocks of 60 trials each: 42 (70%) Go trials, and 18 (30%) No-Go trials. Each trial consisted of the presentation of a black-and-white picture of an adult’s face expressing either happiness or fear, mouth-opened, for 500 ms, separated by 1000 ms inter-stimulus intervals. Faces were selected from a set of 12 models (6 females and 6 males) from the NimStim dataset^38^. In the first block, participants were asked to press Go when they saw a happy face and to withhold responses to a fearful face. The reverse was the case in the second block. Performance in each block and overall was estimated using the sensitivity index *d* prime (ability to discriminate between happy and fear faces), obtained by subtracting the *z*-transformed false alarm rate from the *z*-transformed hit rate.

### Statistical analysis

Differences between the violent offender and control groups in task performance and ECG indices were tested using independent-samples *t*-tests. Correlations between RMSSD at baseline and demographic variables (age, BMI and education levels) were performed using Spearman’s correlations. Correlations across the two groups were compared using z-statistics on the Fisher z-transformed correlation coefficients. The dynamics of RMSSD responsiveness were investigated using repeated measures analysis of variance (ANOVA), in which we examined the effect of group (violent offenders vs. controls), phase (baseline, task, recovery) and their interaction on the standardized values of RMSSD. To explore the effect of psychiatric diagnosis status in the offender group, we repeated this analysis with three groups: Psychiatric offenders, non-psychiatric offenders, and controls. All p-values are of two-sided tests and are reported using false discovery rate correction at *p*_fdr_ < 0.05 to control the overall false positive rate. Uncorrected p-values were reported for exploratory analyses. Effect sizes are reported in terms of Cohen’s *d* and partial eta squared (*η*_*p*_^*2*^).

## Results

### Task performance

Independent samples *t*-tests revealed no significant differences between the groups in performance on the Go Fearful/No-Go Happy face trials (*d* prime MD(se) =  − 0.51(0.26), *t*_df=44_ =  − 1.94, *p*_FDR_ = 0.087, Cohen’s *d* =  − 0.59), the Go Happy/No-Go Fearful face trials (*d* prime MD(se) =  − 0.46(0.31), *t*_df=44_ =  − 1.46, *p*_FDR_ = 0.152, Cohen’s *d* =  − 0.44), or the task overall (*d* prime MD(se) = 0.50(0.25), *t*_df=44_ =  − 2.00, *p*_FDR_ = 0.087, Cohen’s *d* =  − 0.60).

### Preliminary analyses

Table 1 shows the results of independent samples *t*-tests comparing RMSSD in the offender and control groups at the three experimental phases (*p*_FDR_ < 0.05 in bold). The offender group presented significantly lower mean RMSSD during baseline compared to controls. The heart rate values are reported as a general reference and did not differ between groups and phases.

**Table 1:** Mean and standard deviation (SD) of the RMSSD in the offender and control groups.

Variable	Offenders		Controls		*t*	*p*	*p*_*FDR*_^c^	*d*
	Mean	SD	Mean	SD				
RMSSD^a^ (ms^b^) at baseline/rest	25.64	12.63	41.29	19.81	− 3.16	**0.003**	**0.023**	− 0.94
RMSSD (ms) during task	51.14	42.44	57.02	51.69	− 0.42	0.677	0.710	− 0.12
RMSSD (ms) during recovery	35.70	24.78	45.34	21.15	− 1.42	0.162	0.365	− 0.42
Heart Rate (bpm^d^) at baseline/rest	78.67	13.24	74.33	10.27	1.25	0.218	0.392	0.37
Heart Rate (bpm) during task	75.35	11.99	72.51	8.43	0.94	0.355	0.456	0.27
Heart Rate (bpm) during recovery	74.28	11.59	71.04	8.94	1.07	0.293	0.440	0.31

^a^RMSSD = Root mean square of the successive beat-to-beat differences.

^b^ms = millisecond.

^c^FDR = False discovery rate (q = 0.05) to correct for multiple comparisons.

^d^bpm = Beats per minute.

Spearman’s correlations of RMSSD at baseline with age, BMI and level of education were non-significant in either the offender (− 0.27 < r < 0.17, all *ps* > 0.05) or the control (− 0.30 < r < 0.36, all *ps* > 0.05) group. Furthermore, comparing these correlations across the two groups, z-statistic of the Fisher z-transformed correlation coefficients showed no significant differences (all *zs* < 1.84, all *ps* > 0.05).

### RMSSD responsiveness and recovery

**Figure 1:**
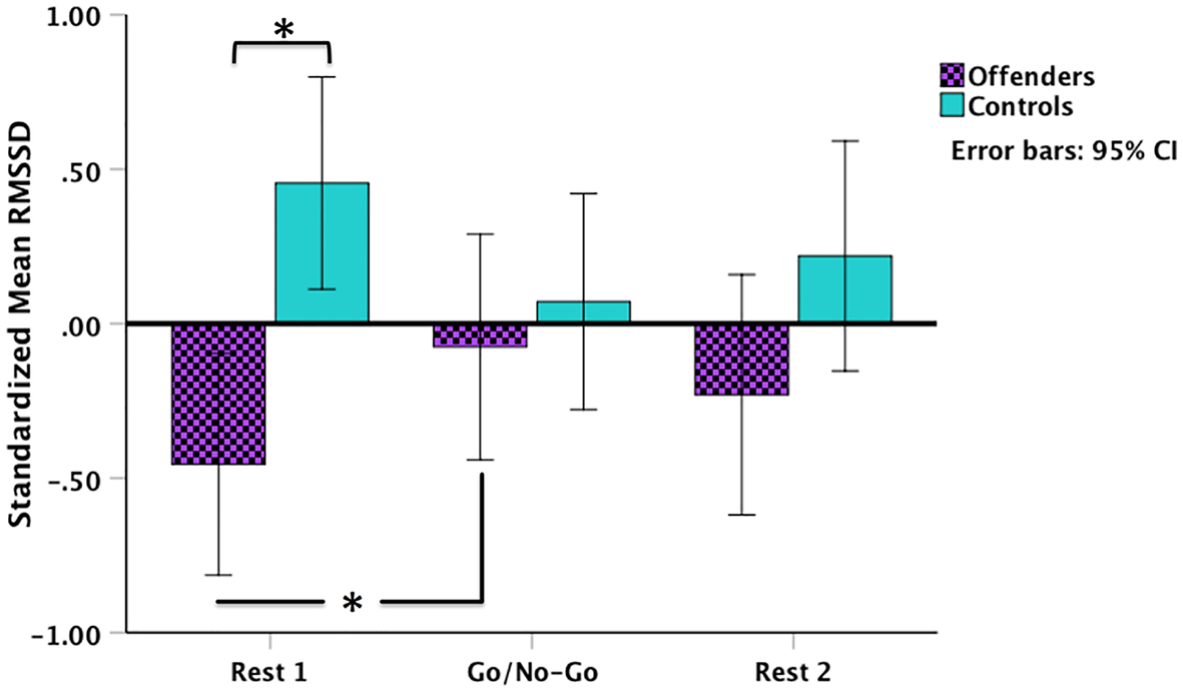
Parasympathetic activity (standardized RMSSD values) in the offender and control groups at baseline (Rest 1), during the emotional Go/No-Go task, and in recovery (Rest 2). Offenders’ RMSSD mean was significantly lower than the controls at baseline, but increased significantly during the task and to a similar level as the controls. *p_*FDR*_ < 0.05.

We observed a significant group by phase interaction (Fig. 1; *F*(2,88) = 5.68, *p* = 0.005, *η*_*p*_^*2*^ = 0.114). Mean RMSSD values of the offender group were significantly lower than the control group at baseline (MD(se) =  − 0.91(0.25), *t*_df=44_ = 3.69, *p*_*FDR*_ = 0.003, Cohen’s *d* =  − 1.11). There were no significant differences between the groups during either the task (MD(se) =  − 0.15(0.25), *t*_df=44_ =  − 0.58, *p*_*FDR*_ = 0.562, Cohen’s *d* =  − 0.18) or recovery (MD(se) =  − 0.45(0.25), *t*_df=44_ =  − 1.68, *p*_*FDR*_ = 0.150, Cohen’s *d* =  − 0.51) phases. Furthermore, while the RMSSD dynamics across the three phases appear to follow curvilinear trends (see Fig. 1), the tests of within-subject contrasts indicated that these were non-significant in either group (Offenders: *F*(1,21) = 4.04, *p* = 0.058, *η*_*p*_^*2*^ = 0.161; Controls: *F*(1,21) = 2.01, *p* = 0.170, *η*_*p*_^*2*^ = 0.080). The linear contrasts were also non-significant (*ps* > 0.05). Moreover, pairwise comparisons revealed a significant increase in parasympathetic activity among the offenders during the task relative to baseline (MD(se) =  − 0.38(0.13), *t*_df=21_ =  − 2.89, *p*_*FDR*_ = 0.027, Cohen’s *d* =  − 1.26). All other comparisons were non-significant (*ps*_*FDR*_ > 0.05).

We repeated the above analysis to explore the association of groups and their interaction with phase, while controlling for RMSSD at baseline. The results of this 2 (offenders vs controls) × 2 (RMSSD task vs RMSSD recovery) repeated measure analysis yielded a nominal effect for group *F*(1,43) = 3.86, *p* = 0.056, *η*_*p*_^*2*^ = 0.082) where the mean RMSSD values of the offender group were lower than the control group (MD(se) = 0.33(0.17), Cohen’s *d* = 0.62). The main effect of phase (*F*(1,43) = 0.001, *p* = 0.978, *η*_*p*_^*2*^ = 0.000) and the group × phase interaction (*F*(1,43) = 0.03, *p* = 0.867, *η*_*p*_^*2*^ = 0.001) were non-significant.

**Figure 2:**
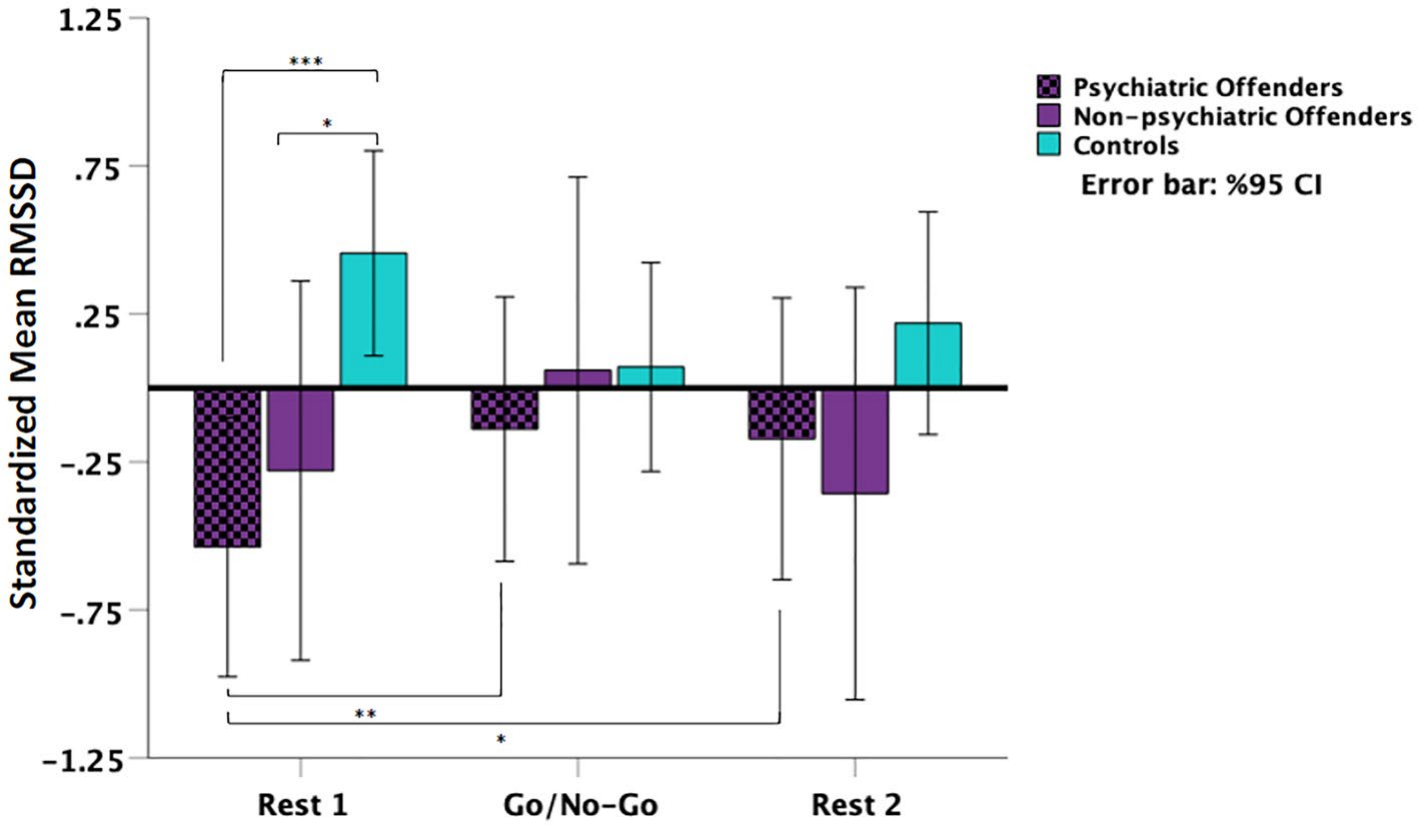
Parasympathetic activity (standardized RMSSD values) broken down by history of psychiatric diagnosis in the offender group compared to controls at baseline (Rest 1), during the emotional Go/No-Go task, and in recovery (Rest 2). Both offender groups, with and without psychiatric diagnoses showed lower RMSSD compared to controls at baseline. In addition, the offenders with psychiatric history showed an increase in their PNS activity during the task compared to baseline, which persisted during recovery. Uncorrected p-values were reported for these exploratory analyses. *p_*uncorrected*_ < 0.05, **p_*uncorrected*_ < 0.01, ***p_*uncorrected*_ < 0.001.

Finally, and in light of the significant results for the HRV data, we wanted to explore if the obtained results were confounded by the presence or absence of a psychiatric diagnosis. To do so, we conducted an additional 3 (psychiatric offenders vs non-psychiatric offenders vs controls) × 3 (RMSSD baseline, RMSSD task, RMSSD recovery) ANOVA. We obtained a significant phase x group interaction (Fig. 2; *F*(4,86) = 3.30, *p* = 0.015, *η*_*p*_^*2*^ = 0.133). Follow up multivariate analysis revealed significant differences between the groups (Pillai’s Trace = 0.357; F(6,84) = 3.05, p = 0.010, *η*_*p*_^*2*^ = 0.179), but only in the mean of the RMSSD at baseline (*F*(2,43) = 6.95, *p* = 0.002, *η*_*p*_^*2*^ = 0.244). Mean RMSSD values of the psychiatric offender group (MD(se) =  − 0.99(0.28), *p*_*uncorrected*_ < 0.001, Cohen’s *d* =  − 1.18) as well as the non-psychiatric offender group (MD(se) =  − 0.73(0.36), *p*_*uncorrected*_ = 0.048, Cohen’s *d* =  − 0.87) were significantly lower than the control group. The mean RMSSD value of the psychiatric offender group was lower but non-significantly different from the non-psychiatric offender group (MD(se) =  − 0.26(0.36), *p*_*uncorrected*_ = 0.506, Cohen’s *d* =  − 0.31). There were no significant differences between the groups during either the task (*F*(2,43) = 0.30, *p* = 0.746, *η*_*p*_^*2*^ = 0.014) or recovery (*F*(2,43) = 1.49, *p* = 0.237, *η*_*p*_^*2*^ = 0.065) phases.

Furthermore, while the RMSSD dynamics across the three phases appear to follow curvilinear trends (see Fig. 2), the tests of within-subject contrasts indicated that these were non-significant in any of the groups (*ps* > 0.05). The linear contrast, however, was significant for the psychiatric offender group (*F*(1,14) = 5.51, *p* = 0.034, *η*_*p*_^*2*^ = 0.283). Moreover, relative to baseline, pairwise comparisons revealed a significant increase in RMSSD mean value among the psychiatric offender group during the task (MD(se) =  − 0.40(0.11), *p*_*uncorrected*_ = 0.003, Cohen’s *d* =  − 0.70), which attenuated by 0.28 SD, but continued to be significant in the recovery phase (MD(se) =  − 0.37(0.16), *p*_*uncorrected*_ = 0.034, Cohen’s *d* =  − 0.42). All other comparisons were non-significant (*ps*_*uncorrected*_ > 0.05).

## Discussion

This study aimed to examine the dynamics of the parasympathetic activity in perpetrators of interpersonal violence before, during, and after an acute laboratory task was performed. As evident by their baseline levels of PNS activity, our findings suggest that offenders’ ANS activity is distinguishable from that of controls. This confirms our first hypothesis, and is consistent with earlier work examining physiological correlates of hostility and aggression^17,20,39^. This distinction is supported by the observation that only four offenders out of the 22 tested obtained RMSSD values above 35 ms at rest, i.e. within 1/2 standard deviation of the expected standard mean of 42 ms^12^. However, our findings do not support our hypothesis that the offender group would be characterized by low parasympathetic responsivity in the task and recovery phases. Contrary to expectations, we observed that their relatively low levels of resting parasympathetic activity rose to levels that were similar to those of the controls during the task, and that their behavioral performance on the task was also equivalent to that of controls. During the recovery phases, while we observed no statistical differences between the groups, or within group differences compared to their respective baselines, there was a tendency for both groups to revert to baseline levels.

From a physiological perspective, we can assume that this low PNS activity at rest could be related to a weakness in the inhibitory functions of the ANS, leading to a persistent hyper aroused state^16,20^. However, the finding of relative increases in PNS activity in the offender group while performing the Go/NoGo inhibition task—to a level similar to controls—is suggestive of flexible use of self-regulatory functions^40^. While caution is warranted in interpreting the results of our study due to the relatively small sample size, we propose that having low levels of PNS activity at rest in the offender group is not indicative of an overall functional impairment in inhibitory control systems per se.

Instead, the offenders were able to mobilize their autonomic resources in a similar way to the controls during the emotional response inhibition task, during which no statistically significant differences in task performance were discerned. This PNS mobilization has implications for understanding mechanisms underlying interpersonal violence and forensic interventions. First, it demonstrates that despite exhibiting low resting HRV—a marker of a tendency towards violence—, offender participants were nonetheless able to respond with autonomic flexibility and engage self-regulatory systems. It remains unknown if increasing baseline levels of PNS activity, for example, through HRV biofeedback training^41,42^, would help to reduce violent reoffending in this group, especially in light of results showing similar levels of Go/No-Go task performance in offenders compared to non-offender controls. Despite the pattern of task performance shown in the current violent offender sample, problems in self-regulation remain a strong predictor of violent crime^43^, and these problems may be most pronounced in certain subgroups of offenders. For example, people whose aggressive acts are better characterized as reactive rather than proactive^44^ may show the most pronounced self-regulatory impairments coupled with a pattern of inflexible physiological responding. Future work should use more person centred approaches (e.g., latent profile analyses) to identify distinct subgroups of adult male violent offenders, based on behavioral and physiological indicators, who are characterised by self-regulatory impairments and who do not show physiological flexibility under cognitive demand. We would suggest that this group of offenders may benefit the most from HRV biofeedback based interventions that aim to increase HRV, and support offenders to exercise self-regulation.

In an exploratory analysis, we also examined differences in physiological responding in the offender group broken down by history of psychiatric diagnosis, compared to non-offending controls. Our results showed that differences between groups were only apparent at baseline, where both offender groups, with and without a history of psychiatric diagnosis, showed lower PNS activity compared to controls. Moreover, the offenders with psychiatric history showed elevated activation of the PNS during the task compared to baseline, and this relative increase persisted during recovery. Our findings are generally in line with earlier work showing reduced baseline levels of HRV in people with a history of psychiatric problems, including anxiety, bipolar, personality, and schizophrenia spectrum disorder (for a review see^45^). The persistence of the task-arousal state into the recovery phase in the psychiatric offender group might be indicative of the perseveration of hypervigilance, possibly due to difficulties in switching-off ANS arousal, as has been described in people with paranoid schizophrenia, for example^46^. The finding that baseline levels of HRV were lower in both offender groups compared to controls supports the conclusion that low resting levels of HRV is associated with increased risk for interpersonal violence independent of the presence of a psychiatric condition. However, caution is warranted in interpreting these findings due to the small size of the groups.

Although our findings provide important insights about PNS dynamics in offenders and non-offenders, future research would benefit from a multisystem approach to the assessment of physiological and autonomic functioning. Like the PNS, the heart is also under the influence of the antagonist pathway SNS, and so measurements including the pre-ejection period and skin conductance reactivity would be revealing about SNS dynamics in these populations. Other measurements including measures of stress reactivity and functioning of the hypothalamic–pituitary–adrenal (HPA) axis would also offer additional perspectives. For example, it has been shown that in boys, but not girls, cortisol reactivity was positively associated with physical aggression but only in those with higher SNS reactivity^47^. These findings highlight the benefits of a multisystem approach, including potential HPA-SNS interactions, and how a multisystem approach may offer greater insight into individual differences in the emergence of behavioral dysregulation^47^.

Our findings suggest that a more comprehensive understanding of the relationship between ANS activity and interpersonal violence requires the implementation of a dynamic approach, whereby ANS activity is assessed at rest, during activity and in recovery. Moreover, the observed pattern of PNS dynamics in offenders is consistent with the suggestion that aggressive and antisocial people can provoke a sense of control that might be necessary when engaging in certain antisocial behaviors, including violent assaults^44^. Accordingly, we hypothesize that while some interpersonal violence might be precipitated by heightened sympathetic, impulsive tendencies, self-regulatory systems might nonetheless be recruited while performing the act of violence.
